# Prospective Spatiotemporal Cluster Detection Using SaTScan: Tutorial for Designing and Fine-Tuning a System to Detect Reportable Communicable Disease Outbreaks

**DOI:** 10.2196/50653

**Published:** 2024-06-11

**Authors:** Alison Levin-Rector, Martin Kulldorff, Eric R Peterson, Scott Hostovich, Sharon K Greene

**Affiliations:** 1 Bureau of Communicable Disease New York City Department of Health and Mental Hygiene Long Island City, NY United States; 2 Information Management Services, Inc Calverton, MD United States

**Keywords:** communicable diseases, disease outbreaks, disease surveillance, epidemiology, infectious disease, outbreak detection, public health practice, SaTScan, spatiotemporal, urban health

## Abstract

Staff at public health departments have few training materials to learn how to design and fine-tune systems to quickly detect acute, localized, community-acquired outbreaks of infectious diseases. Since 2014, the Bureau of Communicable Disease at the New York City Department of Health and Mental Hygiene has analyzed reportable communicable diseases daily using SaTScan. SaTScan is a free software that analyzes data using scan statistics, which can detect increasing disease activity without a priori specification of temporal period, geographic location, or size. The Bureau of Communicable Disease’s systems have quickly detected outbreaks of salmonellosis, legionellosis, shigellosis, and COVID-19. This tutorial details system design considerations, including geographic and temporal data aggregation, study period length, inclusion criteria, whether to account for population size, network location file setup to account for natural boundaries, probability model (eg, space-time permutation), day-of-week effects, minimum and maximum spatial and temporal cluster sizes, secondary cluster reporting criteria, signaling criteria, and distinguishing new clusters versus ongoing clusters with additional events. We illustrate how to support health equity by minimizing analytic exclusions of patients with reportable diseases (eg, persons experiencing homelessness who are unsheltered) and accounting for purely spatial patterns, such as adjusting nonparametrically for areas with lower access to care and testing for reportable diseases. We describe how to fine-tune the system when the detected clusters are too large to be of interest or when signals of clusters are delayed, missed, too numerous, or false. We demonstrate low-code techniques for automating analyses and interpreting results through built-in features on the user interface (eg, patient line lists, temporal graphs, and dynamic maps), which became newly available with the July 2022 release of SaTScan version 10.1. This tutorial is the first comprehensive resource for health department staff to design and maintain a reportable communicable disease outbreak detection system using SaTScan to catalyze field investigations as well as develop intuition for interpreting results and fine-tuning the system. While our practical experience is limited to monitoring certain reportable diseases in a dense, urban area, we believe that most recommendations are generalizable to other jurisdictions in the United States and internationally. Additional analytic technical support for detecting outbreaks would benefit state, tribal, local, and territorial public health departments and the populations they serve.

## Introduction

### Background

The Bureau of Communicable Disease (BCD) at the New York City (NYC) Department of Health and Mental Hygiene monitors electronic reports daily of >70 reportable infectious diseases for an urban population of approximately 8.5 million residents [1]. Since 2014, to help prioritize resources for case and outbreak investigations and response activities, the BCD has automated daily analyses to prospectively detect and monitor spatiotemporal clusters of reportable communicable diseases using SaTScan [2,3]. SaTScan (an abbreviation of *Space and Time Scan Statistics*) is a free software that analyzes data using scan statistics [4], which can detect increased disease activity without a priori specification of temporal period, geographic location, or size. SaTScan has effectively detected clusters of enteric and respiratory diseases and of antimicrobial-resistant infections using varied data sources from settings worldwide [5-11]. The BCD has applied several prospective SaTScan analyses to address varied surveillance needs in NYC, with early detection and near–real-time monitoring of salmonellosis, legionellosis, shigellosis, and COVID-19 outbreaks [2,12-14]. For example, in 2015, SaTScan analyses provided the first signal for the second largest outbreak of community-acquired legionellosis in the United States [13]. Detecting serious outbreaks even a day or 2 earlier can potentially save lives.

In the absence of centralized training and technical support for designing and fine-tuning such systems, we have provided informal consultations for other health departments. While there are scientific papers describing the statistical methods [4,15-19] and the SaTScan user guide [20] explains input data requirements and various software features, this is the first such practical resource for health department staff.

### Objectives

We aimed to share our recommendations for health departments to design and fine-tune a system to detect community-acquired reportable communicable disease outbreaks using SaTScan (Tables 1-3). This guide describes requirements and parameter settings for a variety of analytic aims and how to use built-in features to interpret results. Methods for detecting building-level outbreaks, hospital-associated outbreaks, purely temporal clusters such as seasonal increases, or early warning signs of diseases with pandemic potential are described elsewhere [21-24].

**Table 1:** Summary of input file and parameter setting recommendations for designing a system to detect reportable communicable disease outbreaks using SaTScan.

Section and subtopic		Recommendations
Input data		1. The case file for each reportable disease should contain the census tract of the patient home address and the best approximation of illness onset date and can contain additional patient information such as gender and age. 2. Include all reported events in the case file, whether subsequently confirmed or not. 3. The coordinates file should include 1 row per census tract with its latitude and longitude. 4. A network file can be used to avoid clusters that span hard-to-cross boundaries, such as lakes, rivers, and mountain ranges.
**Parameter settings**		
	Analysis type	5. Use prospective space-time analysis.
	Probability model	6. Use the space-time permutation probability model. 7. If case ascertainment over both space and time is highly affected by testing variability, the Bernoulli or Poisson model may be used instead. This additionally requires a control file of negative tests or a population file of all tests.
	Spatial and temporal adjustments	8. The space-time permutation model automatically adjusts for purely spatial variations and for purely temporal patterns. 9. Adjust for day-of-week by space interaction.
	Minimum number of cases	10. To quickly detect outbreaks before many events accrue, use the default minimum number of events of 2. 11. To support resource allocation to areas with high and increasing disease transmission rather than prioritize case investigations, the minimum number of events can be increased.
	Maximum spatial cluster size	12. For most jurisdictions, allow the cluster to expand in size to include up to 50% of all events during the study period. 13. For geographically large jurisdictions, either use 10% as the maximum cluster size or add another maximum cluster size (eg, 200 km).
	Temporal cluster size	14. For most communicable diseases, scan for clusters with a minimum of 2 days and a maximum of 30 days.
	Study period	15. The study period should preferably be at least 3 times as long as the maximum temporal cluster size. A study period of 1 year is usually reasonable. 16. The end of the study period should be set to previous day or the most recent day with sufficiently complete data.
	Time aggregation	17. Aggregate to daily resolution.
	Frequency of analysis	18. Run analyses daily.
	RIs^a^ and inference	19. Use the *default P value* method with the maximum number of Monte Carlo replications set to 999. 20. Use a signaling threshold of RI≥100 days. 21. Consider an RI of 100 to <365 days as a weak cluster, an RI of 365 days to <5 years as a moderate cluster, an RI of 5 to <100 years as a strong cluster, and an RI of ≥100 years as a very strong cluster.
	Secondary clusters	22. Enable the *Most Likely Clusters, Hierarchically* option and choose *No Cluster Centers in Other Clusters*.

^a^RI: recurrence interval.

**Table 2:** Summary of output files and built-in feature recommendations for cluster interpretation when using SaTScan to detect reportable communicable disease outbreaks.

Section and subtopic			Recommendations
**Output files and built-in features**			
	Text-based results file	1. Review information about the detected clusters and the analysis performed.	
	Line list of cluster events	2. Review line list of events in clusters exceeding the RI^a^ signaling threshold. 3. Distinguish events that are newly added to ongoing clusters by using an events cache input file. 4. Inspect the line list for data quality problems that may have been missed by other routine checks, such as duplicated events in the case file, misreported patient addresses, or addresses unrelated to possible exposure sites. 5. Inspect the line list to determine whether the cluster affects multiple households and suggests community transmission vs primarily affects persons in the same household or building.	
	Map	6. Visualize the spatial extent of clusters. 7. Observe patterns of event locations and group by any chosen variable included in the case file.	
	Temporal graphs	8. Visualize observed and expected event counts inside and outside the geographical cluster area, marking the period before and during the cluster.	
	Drilldown analysis	9. Conduct a drilldown analysis if you wish to determine whether events in a cluster are randomly distributed within that cluster. 10. In parallel, consider rerunning the analysis applying a maximum reported spatial cluster size as both approaches may detect significant clusters within larger clusters.	

^a^RI: recurrence interval.

**Table 3:** Summary of recommendations for assessing and fine-tuning a system to detect reportable communicable disease outbreaks using SaTScan.

Section and subtopic			Recommendations
**System assessment**			
	Proof of concept	1. Identify at least 1 known historical acute, localized, community-acquired outbreak and mimic prospective surveillance around that period to assess the speed and precision with which the outbreak could have been detected.	
	System performance	2. If the system consistently produces clusters that are too large or too delayed to be useful, misses signals, or produces too many false or weak signals, first examine data quality by inspecting input files and line list output files for data errors before fine-tuning the system.	
**System fine-tuning when outbreak detection is delayed or missed**			
	Difficulty detecting geographically small outbreaks or outbreaks at the boundaries of geographic units	3. Disaggregate data into smaller geographic units.	
	Difficulty detecting outbreaks affecting a particular age group	4. Conduct additional age-restricted subgroup analyses, such as for children aged <5 years.	
	Difficulty detecting outbreaks in which people are exposed far from their home	5. Collect and analyze multiple addresses per patient, such as both home and work.	
	Difficulty detecting outbreaks because of missing data	6. Conduct quality assurance to detect and resolve drop-offs in laboratory reporting by laboratory and disease. 7. To avoid missing outbreaks affecting unsheltered patients experiencing homelessness and without a geocodable address, assign them to an artificial census tract.	
	Difficulty detecting new outbreaks in areas with previous outbreaks	8. From the baseline period in the case file, exclude days during major previous outbreaks. 9. For previous outbreaks limited to a specific building or institution, exclude those events from the baseline period in the case file.	
	Difficulty detecting outbreaks affecting people who reside in areas not connected in the network file	10. Add more connections to the network file.	
	Difficulty detecting weak clusters that are potentially actionable outbreaks	11. If using an RI^a^ threshold for signaling of ≥1 year, decrease the threshold.	
**System fine-tuning when consistently finding large, uninteresting clusters**			
	Clusters span hard-to-cross boundaries, such as lakes, rivers, and mountain ranges	12. Use the network input file.	
	Clusters with a relative risk near 1 are of limited public health interest	13. Impose a minimum relative risk restriction.	
**System fine-tuning when there are too many signals**			
	Clusters are driven by duplicate events or incorrect patient addresses	14. Proactively check for and correct data errors.	
	Clusters are driven by changing nature of input data, such as recent adoption of culture-independent diagnostic testing	15. Shorten the baseline study period.	
	Clusters are driven by reports that do not represent true illnesses, such as reports of patients with only negative laboratory test results	16. Use stricter disease definition inclusion criteria and impose a time lag to allow for investigating and ruling out cases.	
	Clusters are driven by within-household transmission, so they do not represent community transmission	17. Retain only 1 event per household.	
	Clusters represent weak outbreaks that are not actionable given available resources	18. If using an RI threshold for signaling of <1 year, increase the threshold.	

^a^RI: recurrence interval.

### Organization

Providing context and examples to build intuition, we discuss system setup requirements, data quality assurance, input file preparation, parameter setting recommendations derived from public health and statistical principles, and new features in the 2022 SaTScan release that simplify routine analyses. Multimedia Appendix 1 [2,13,14,25-37] provides further details about parameter settings and advanced options, demonstrates cluster interpretation using artificial but realistic data, and offers strategies for fine-tuning analyses. In Multimedia Appendix 2, a video demonstrates how to set up a cluster detection system, and sample input and output files are provided.

## Analysis Design

### Ethical Considerations

The BCD’s prospective cluster detection activity was deemed nonresearch, public health surveillance by the NYC Department of Health and Mental Hygiene Institutional Review Board. Figures in this tutorial are for illustrative purposes only and reveal no confidential patient information.

### Requirements and System Setup

A strong informatics infrastructure and uninterrupted data streams are key for a successful prospective reportable disease outbreak detection system. Near–real-time electronic laboratory reporting with patient location data is required, as well as epidemiologists to interpret signals and resources to investigate outbreaks.

Local installation of SaTScan is needed, and confidential information is never transmitted elsewhere. Google Earth installation and mail server information are optional requirements for certain SaTScan features.

To ensure optimal system performance, we recommend proactive data quality assurance practices, including monitoring for data feed interruptions, deduplicating events reported more than once, and correcting patient information (eg, correcting typographical errors in addresses failing to geocode, correcting when laboratories misreport patients’ residential addresses, maintaining geocoding reference files to account for newly constructed buildings, and correcting implausible onset dates).

SAS or R can be used to prepare input data, run SaTScan in batch mode, create output summary files and visualizations, and send email alerts. Sample code for these systems is freely available [2,38-40], but the 2022 SaTScan release eliminates the need for much of this complicated, external code.

### Defining the Aim

Most routine prospective SaTScan analyses of reportable communicable diseases are designed to quickly detect and monitor spatiotemporal outbreaks using patients’ home addresses [2]. We refer to this as our *base analysis*. In these disease-specific analyses, cluster summaries and patient line lists are routed to in-house disease experts for interpretation and potential action to stop transmission or to focus public health resources. We believe this system is relevant for all acute infectious diseases except for diseases of which a single case is a public health emergency, such as anthrax or botulism. For a few diseases, we modified the base analysis (Table S1, Figure S1, and the Analysis Design: Supplement section in Multimedia Appendix 1).

### Input Data

#### Overview

SaTScan detects data aberrations, which can represent true clusters or data errors. Hence, input data require attention to detail. A case file contains all disease events with their epidemiologically relevant location (eg, census tract of home address) and date (eg, illness onset date), and a coordinates file includes all locations (eg, census tracts) together with the latitude and longitude of their center points. We refer throughout to *events* as all reported disease events and *cases* as the subset that meet the surveillance case definitions established by the US Centers for Disease Control and Prevention and the Council of State and Territorial Epidemiologists [41].

#### Case File

The case file must contain a location ID (eg, census tract number), a temporal element such as a date, and the number of events at that location and time. The case file may be aggregated by location and date. Alternatively, it can be formatted as having 1 row per event and may contain additional columns that are not used in the analysis but can be output in a line list or used to group events on a map (refer to the Built-In SaTScan Implementation and Output Interpretation Features section).

For our base analyses, the temporal element is the best approximation of the date when patients became ill, prioritizing symptom onset date, diagnosis date, or report date such that there is no missingness in the temporal data element. The spatial location is the census tract of patient residence. Some degree of spatial aggregation is necessary to scan along a network (refer to the Network File section), and geocoding to census tracts is sufficiently fine resolution. Even if the public health action occurs at a larger spatial unit, using smaller geographic units increases both the precision and statistical power to quickly detect emerging outbreaks, including those that are geographically small or that do not conform to the boundaries of larger administrative areas. Patients who have a missing, inaccurate, or undefined spatial location can either be purposely excluded from the analysis or included by assigning them a location (eg, assigning patients whose home address is a post office box to the census tract of the post office holding their mail and assigning unsheltered patients experiencing homelessness to a unique, artificial census tract).

For some analyses, we restrict the case input file. For amebiasis, cryptosporidiosis, giardiasis, and shigellosis, we analyze all ages combined as well as children aged <5 years separately to detect outbreaks that might affect patients attending childcare programs. For diseases for which household transmission is likely, for example, norovirus and shigellosis, we restrict the case input file to include only 1 event per household (refer to the Difficulty Detecting Outbreaks Affecting a Particular Age Group and Clusters Are Driven By Within-Household Transmission sections in Multimedia Appendix 1).

If there is a spatiotemporal outbreak in the baseline, it may become harder to prospectively detect a subsequent outbreak. If a purely spatial adjustment is applied, either explicitly or implicitly as in the space-time permutation model, then it will be harder to detect subsequent outbreaks around the same location. This problem can be resolved by removing historic outbreaks from the input files (refer to the Difficulty Detecting New Outbreaks in Areas With Prior Outbreaks section in Multimedia Appendix 1).

We include all reported events during the study period specified in the parameter settings regardless of whether they are subsequently confirmed according to Centers for Disease Control and Prevention and Council of State and Territorial Epidemiologists surveillance case definitions [41] upon investigation. By analyzing all reported events, timeliness is preserved in that events can be analyzed as soon as they are reported rather than waiting until investigation and case classification processes are complete. Ensuring consistent event inclusion criteria across the study period supports a valid comparison of total reporting volume between current and historical periods [42].

#### Coordinates File

The coordinates file has 1 row per location with a location ID and its geographic coordinates. Most BCD analyses use the latitude and longitude of each census tract centroid per census 2010 definitions.

Alternatively, *space* need not be conceptualized as geographical coordinates. To identify temporal clusters of infections by particular *Salmonella* serotypes, we replace *space* with arbitrary coordinates for serotype and detect citywide clusters of particular serotypes that could not be explained by the overall seasonality of *Salmonella* infections [25].

#### Network File

Spatial scan statistics can be used with windows of different shapes, including circular, elliptical, and nonparametric [43-46]. SaTScan is typically used with a circular scanning window, which also has very good statistical power to detect clusters of other geographical shapes and sometimes better power to detect irregularly shaped clusters than flexibly shaped spatial scan statistics [47]. This is because flexibly shaped spatial scan statistics without some form of noncompactness penalty will often detect spindly, octopus-like clusters that cherry-pick areas with high rates and with long, thin connections between them [47].

**Figure 1:**
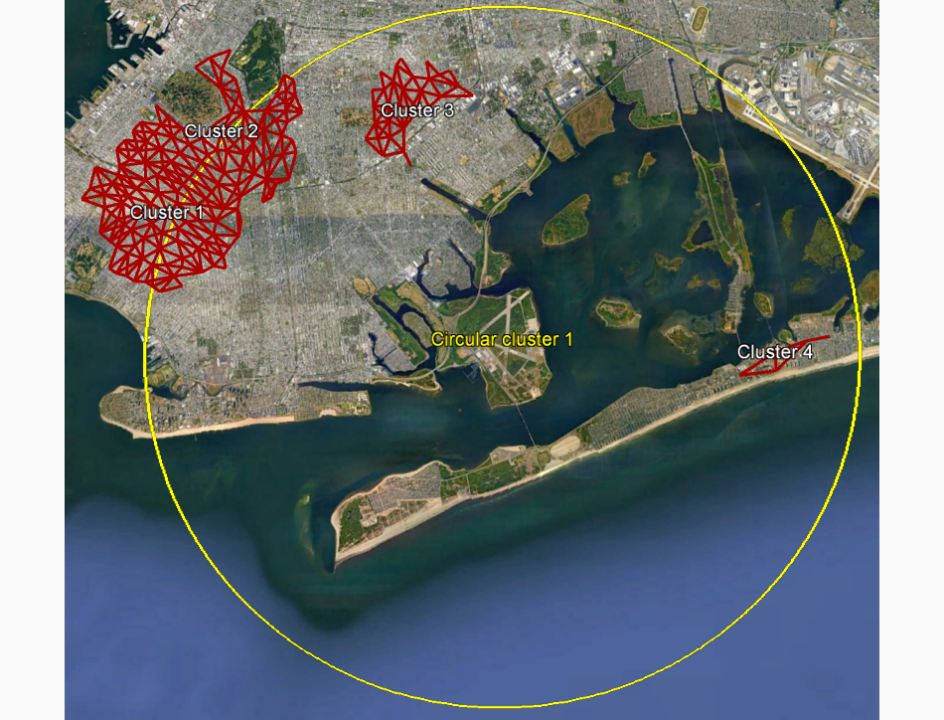
Illustrative cluster detected using a circular scanning window (in yellow) versus scanning along a network (in red) using the same input data. Scanning along a network accounts for limited connectivity across water bodies, resulting in more precise spatial clusters, such as illustrative cluster 4 in the Rockaway peninsula in Queens, New York City.

An enhancement in SaTScan version 10.0 is the option to scan locations along a network, which may represent travel distance between locations or the amount of interaction between different communities. For example, census tracts or zip code tabulation areas may be geographically close but separated by lakes, rivers, or mountain ranges. We recommend constructing a network file connecting neighboring locations unless separated by those barriers so that clusters can form around rather than through them (Figure 1; refer to Figure S2 and the Network File: Supplement section in Multimedia Appendix 1).

### Parameter Settings

Unlike machine learning methods, in which hyperparameters are tuned using real data during model development, SaTScan parameter settings are derived from underlying public health and statistical principles that should be generalizable across jurisdictions.

#### Type of Analysis and Probability Model

We use the prospective space-time permutation scan statistic for almost all routine SaTScan analyses conducted at the BCD, including the base analysis [2,17,25]. Prospective analyses detect currently active unusual clusters, evaluating only the subset of possible clusters that encompass the last day of the study period together with a flexible number of previous days. Cluster statistics are determined by the counts of observed and expected events within a cluster window, which is bounded by the user-specified minimum and maximum temporal cluster size. For the space-time permutation scan statistic, the expected number of events for any area is a function of the observed counts during the baseline period and the jurisdiction-wide trend during the temporal window [17]. For example, if a collection of 5 census tracts has 2.3% of cases during the baseline period and there are 100 total cases in the jurisdiction during the cluster period, then the expected number of cases during the cluster period in those 5 census tracts is 2.3.

SaTScan compares a statistical likelihood function calculated from the real data sets with likelihoods calculated from random data sets generated under the null hypothesis of no clustering. There is a cluster if a geographical area has a higher proportion of its events in the cluster period compared with the previous baseline period or, equivalently, if a period has a higher proportion of its events within versus outside the geographical cluster area. For example, if Manhattan’s Upper East Side has 10% of all NYC events during the last 7 days but only 3% of all events during the previous months, a prospective space-time permutation analysis will detect a cluster in the Upper East Side during the most recent week. Note that, while such a cluster is most likely due to an outbreak or increased testing in the Upper East Side, it can also be generated by a sudden decrease in disease occurrence or ascertainment in the rest of the city. The former is more likely for geographically small clusters, whereas larger clusters could have either explanation.

The *permutation* in the *space-time permutation* model refers to keeping all observed locations and dates fixed when simulating random data sets under the null hypothesis while randomly shuffling which observed event locations are connected to which observed dates. By conditioning on locations and dates with observed events, the space-time permutation model automatically adjusts nonparametrically for any purely temporal patterns, including seasonality, secular trends, and day-of-week effects, as well as any purely geographical variations in disease occurrence, diagnosis, and reporting, such as due to insurance coverage and access to care. An area’s larger population size, which also affects disease incidence, is accounted for by having more disease counts observed in the baseline period.

For count data such as disease events, Bernoulli and Poisson models are also available. However, the space-time permutation model is often preferred because it requires only event data and automatically adjusts nonparametrically for both purely temporal and spatial variations (refer to the Bernoulli and Poisson Probability Models section and Figure S1 in Multimedia Appendix 1).

#### Space and Time Adjustments

If there is an overall increasing temporal trend, SaTScan may identify an outbreak if there is no temporal adjustment. One may want to adjust for naturally occurring seasonal variation, for example, while still detecting local outbreaks not explained by jurisdiction-level seasonal trends.

When applying a spatial adjustment, SaTScan will identify clusters in which case counts are increasing faster or decreasing slower than elsewhere in the jurisdiction even if the absolute level is lower than that in surrounding areas.

The space-time permutation model automatically adjusts for purely temporal patterns and purely spatial variations, unlike the Bernoulli and Poisson models, for which these adjustments must be explicitly requested. We additionally adjust for space by day-of-week interaction in our base analysis because the day-of-week pattern of health care–seeking behavior and diagnoses may vary geographically in NYC. With this adjustment, the dates in which events occurred are shuffled and assigned to the original event locations, restricting them to the same day of the week. This adjustment should only be applied when there are several weeks in the study period; with only a few weeks, the day-of-week–specific estimates become too unstable. We do not use covariate adjustment because this is unnecessary for prospective outbreak detection and doing so could adjust away emerging outbreaks affecting a particular demographic group.

#### Scanning for High or Low Rates

Most BCD analyses scan for areas with high rates as we are interested in detecting outbreaks of excess events. The one exception is data quality analyses, where we scan for low rates to quickly identify laboratories with unusually low reporting volume [26].

#### Minimum Number of Events

In the base analysis, we require only 2 events at minimum because we want to quickly detect outbreaks before many events accrue. For example, 2 salmonellosis events on the same day residing on the same block could be of great interest. For COVID-19, we set the minimum number of events in a cluster to 100 when volume was high because the purpose was to support resource allocation to areas with high and increasing disease transmission rather than to conduct case investigations and contact tracing to identify a common exposure source.

#### Maximum Spatial Cluster Size

The option that imposes the fewest assumptions is to allow the cluster to expand in size to include up to 50% of all events during the study period. This is reasonable for modestly sized areas such as NYC but might yield too large and uninformative clusters in national- or state-level jurisdictions. For geographically large study areas, we recommend either using 10% as the maximum geographical cluster size or adding another maximum geographical cluster size. For example, a maximum of 50% may appropriately limit the cluster size around Anchorage, whereas Western Alaska may be better served with a 200-km maximum cluster radius. When using a network file and defining a geographical maximum spatial cluster size, a cluster will expand until reaching a network location at the specified distance from the cluster centroid, which allows for irregularly shaped clusters. We recommend erring on the side of searching for larger clusters, followed by using other principled approaches to interpret or prevent large clusters (refer to the System Fine-Tuning section in Multimedia Appendix 1).

The BCD has on occasion applied a maximum spatial cluster size of a 1-km radius. To search for clusters of locally acquired Zika virus infection, we assumed that a maximum 1-km radius was consistent with the typical flight distance of an *Aedes* mosquito [27] plus some additional distance to include residences of people moving around and coming into contact with the same infected mosquito. To search for clustering of legionellosis events around cooling towers to guide environmental sampling [13], we considered a 1-km radius to be consistent with the area of highest risk of exposure around a contaminated cooling tower.

Of note, setting the maximum spatial cluster size is different from applying a maximum reported spatial cluster size in the advanced output features, which limits the clusters *reported* in the output rather than limiting the clusters *evaluated* by the analysis. While it is OK to vary the maximum reported spatial cluster size, an analysis should never be repeated with a different maximum spatial cluster size as *P* values and recurrence intervals (RIs) are inaccurate if one manipulates and tries multiple such parameter settings [48].

#### Minimum and Maximum Temporal Cluster Size

The base analysis scans for clusters that are between 2 and 30 days long, which is enough to encompass the upslope of an epidemic curve for point-source outbreaks, typically spanning a few days or weeks depending on the pathogen. We extended the maximum temporal cluster size to 60 days for salmonellosis, Shiga toxin–producing *Escherichia coli*, paratyphoid fever, and typhoid fever and to 120 days for listeriosis to align with PulseNet USA definitions based on clustering in time and genetic relatedness [49]. Although campylobacteriosis, shigellosis, noncholera *Vibrio spp.* infection, and yersiniosis are also PulseNet organisms, we kept the maximum temporal cluster size at 30 days to slightly increase power to detect more focal clusters of these diseases. These diseases are higher volume and commonly detected using culture-independent diagnostic testing such that laboratory subtyping is not routinely available in NYC to support defining clusters. We similarly extended the maximum temporal cluster size to 60 days for hepatitis A because the incubation period can extend to 50 days [50] and to allow additional time for patient care seeking.

A maximum of 30 or 60 days may not be long enough for diseases with long incubation periods, extended propagated transmission, or intermittent common-source outbreaks. For example, exposure to point sources of listeriosis can be intermittent given persistent environmental contamination, and the incubation period can be nearly 70 days [28], so for this disease, we scan for clusters between 7 and 365 days at weekly aggregation.

In the base analysis, we set the minimum temporal cluster size to 2 days because few true outbreaks of the diseases we monitor span only 1 day, and not evaluating clusters of only 1-day duration slightly improves power given less statistical adjustment for multiple comparisons. However, note that a 2-day cluster can have all cases on the same day. If there will be no public health action unless a cluster has persisted for some time, the minimum temporal cluster size can be lengthened. For COVID-19, for example, we scanned for clusters with a minimum temporal size of 14 days to support resource reallocation to areas with sustained high test positivity.

#### Study Period

It is important to balance the need for a period long enough to establish a stable local baseline for each spatial unit yet short enough to avoid variable secular trends due to, for example, geographical population shifts over time. As a rule of thumb, the study period should preferably be at least 3 times as long as the maximum temporal cluster size. This is analogous to a case-control study, which has diminishing returns in statistical power when the control-to-case ratio is increased beyond approximately 3 [51]. If there is a permanent change in surveillance so that recent and older data are no longer comparable, the study period should be shortened. For example, laboratories adopting a culture-independent diagnostic test can lead to increased case ascertainment, and temporarily shortening the study period to begin after the new test was adopted can restore consistency in the baseline [29] (refer to the Clusters Are Driven by the Changing Nature of Input Data section in Multimedia Appendix 1).

For timeliness, the end of the study period should be set to the previous day or the most recent day with sufficiently complete data. In the base analysis, the study period is 1 year with an end date set to the day before the analysis is run. Although 1 year is >3 times longer than the maximum temporal cluster size of 30 days, case ascertainment is generally consistent over a 1-year period for most diseases, and the longer study period provides a more stable baseline. For COVID-19, we shortened the study period to 63 days because volatility in testing access and outreach during the public health emergency made older data less comparable, and we set the end of the study period to 3 days before the run date because more recent testing data were largely missing [14].

#### Time Aggregation

With thousands of locations, long study periods, and multiple data streams, space-time SaTScan analyses can be computationally intensive. To reduce computing time, data may be aggregated into longer time intervals by, for example, aggregating daily data into weekly data. Another reason for aggregating to time intervals of 7 days is to remove day-of-week effects. For the base analysis, we use a time aggregation of 1 day to detect outbreaks as quickly as possible, increase the statistical power to detect outbreaks that do not conform to weekly or other prespecified time intervals, and obtain a more precise estimate of the cluster start date.

#### Frequency of Analyses

With near–real-time electronic laboratory reporting, we run analyses daily. If prospective analyses are conducted at a different frequency than the aggregated time units, such as weekly analyses with daily data, the analysis frequency must be specified. This may be needed if data are reported weekly but with daily resolution; if weekly analyses are sufficiently frequent given limited staffing capacity; or to support public health objectives other than acute outbreak investigations, such as guiding resource allocation. If it is important to monitor for unusual clusters near continuously (eg, hourly analyses to detect early indications of a possible bioterrorist attack), consider inputting data using the generic time precision option.

#### Secondary Clusters

Any disease may have multiple active outbreaks at any moment, so secondary clusters should be reported by enabling the *Most Likely Clusters, Hierarchically* option. We use the *No Cluster Centers in Other Clusters* option rather than the default *No Geographical Overlap*. This allows for reports of slightly overlapping clusters so that clusters located close to each other can be detected and can also help define the extent of irregularly shaped outbreaks. At the same time, it avoids redundant clusters that are almost identical to each other as well as clusters almost completely driven by the primary cluster but with large outlying areas with no or modest excess risk. Epidemiological judgment must be used to determine whether events in overlapping clusters are attributable to a single or multiple outbreaks or whether the secondary cluster is of limited interest. It may not be worthwhile to investigate every event in a large secondary cluster with a much lower RI that has a large proportion of events that are also in the primary cluster.

#### RIs and Inference

Monte Carlo hypothesis testing compares the maximum likelihood value for the real data with the maximum likelihoods from each of the random replicas of the data set. For prospective analyses, SaTScan assigns an RI to each cluster. The RI is an alternative to the *P* value (RI=1/*P* value), and the greater the RI, the less likely the cluster is due to chance. If a cluster has an RI of 1 year, then under the null hypothesis and during any 1-year period, the expected number of false signals with the same and higher magnitude is 1. For rare diseases, this expected number is lower depending on cluster size restrictions. While *P* values are commonly used for determining statistical significance, the typical cutoff of *P*<.05 is not meaningful for prospective analyses. With daily analyses for 1 disease, applying a *P*<.05 threshold would generate 1 false signal every 20 days on average, or approximately 18 false signals per year, which is too frequent.

Increasing the number of Monte Carlo replications generating random data sets under the null hypothesis slightly increases the statistical power of the scan statistic but also increases the run time. Our base analysis uses 999 replications, with the *default P value* option to end early if the *P* value is large. The number of replications must be at least 999 to avoid unnecessary loss of power.

#### Signaling Threshold

In the base analysis, we set the signaling threshold to RI≥100 days. We think of a cluster with an RI of 100 to <365 days as a weak cluster, a cluster with an RI of 365 days to <5 years as a moderate cluster, a cluster with an RI of 5 to <100 years as a strong cluster, and a cluster with an RI of ≥100 years as a very strong cluster. *P* values or RI thresholds determine which clusters trigger an alert, but they should be considered alongside other factors to establish whether a cluster is of public health importance [52]. While we have suggested rules of thumb for RI interpretation, investigators should holistically interpret other cluster characteristics and apply epidemiological judgment considering the disease severity, relative risk, location, period, and patient characteristics in the line list (refer to the Cluster Investigation and Response section).

### Assessment of Analysis Design

Our SaTScan parameter settings are derived from underlying public health and statistical principles. While we believe that our recommendations are generalizable, parameter settings may require adjustment for other diseases and jurisdictions. When using real-world rather than simulated data, there is generally no gold standard that can be used to evaluate results. For local proof of concept, one way to determine whether an analysis needs adjustments is to identify at least one known historical acute, localized, community-acquired outbreak and mimic prospective surveillance around that period to assess the speed and precision with which the outbreak would have been detected.

If an analysis consistently produces results that are unsatisfactory and data quality issues have been ruled out as a cause, then the input files or parameter settings may need fine-tuning. Issues we have encountered include missed or delayed outbreak detection, clusters too large to be useful, and too many signals to be actionable given available resources (refer to the System Fine-Tuning section in Multimedia Appendix 1).

## Built-In SaTScan Implementation and Output Interpretation Features

SaTScan version 10.1 introduced several built-in features to simplify adopting and implementing routine prospective analyses and streamline output interpretation. These include a tool for automating multiple prospective analyses, temporal and geographical visualizations of clusters and events, line lists with information about cluster events, and a feature that allows users to send automated email alerts summarizing analysis results (refer to the Cluster Output Interpretation section in Multimedia Appendix 1 and video demonstration in Multimedia Appendix 2).

### Multiple Analyses

When independently monitoring multiple diseases, running multiple analyses is necessary. This is easily managed using the multiple analyses feature. The only preanalysis coding required is to generate the input files in the appropriate format (refer to the video demonstration in Multimedia Appendix 2).

### Temporal Graphs

**Figure 2:**
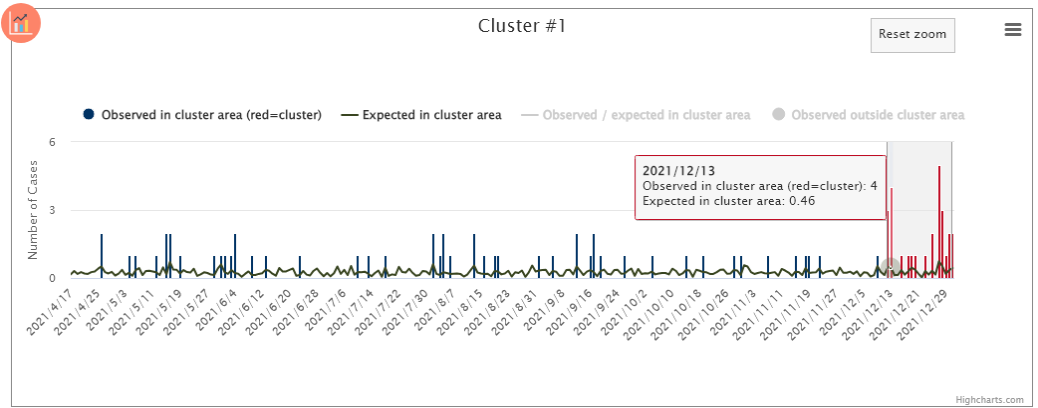
SaTScan-generated temporal graph depicting the observed and expected event counts in the geographical area of the cluster during (band shaded in gray) and before the cluster temporal window.

Temporal graphs are useful for visualizing epidemic curves. SaTScan can produce temporal graphs that display observed and expected event counts inside and outside the geographical cluster area, marking the period before and during the cluster (Figure 2; refer to the Temporal Graphs: Supplement section in Multimedia Appendix 1).

### Visualizing Clusters and Cases on a Map

SaTScan can produce maps for visualizing the spatial extent of clusters in HTML, KML, and shapefile formats. Locations of events during the study period can be added to the map and can be grouped by any variable (eg, case status, age group, and gender), distinguished using different icons.

**Figure 3:**
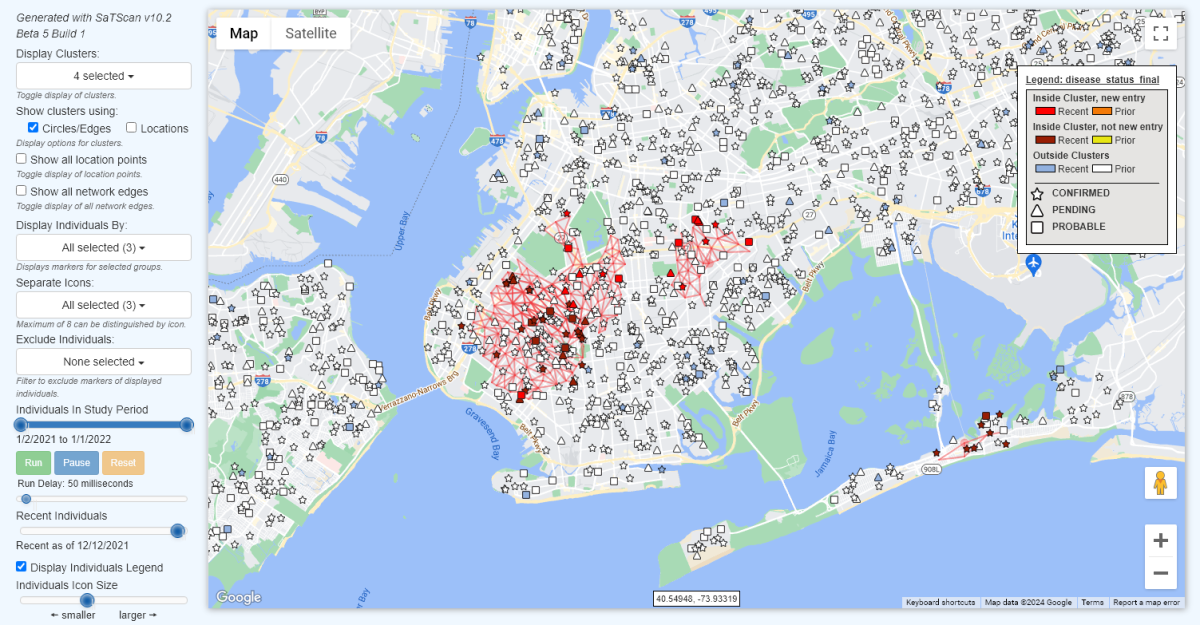
HTML visualization produced by SaTScan depicting 4 clusters exceeding the signaling threshold with (artificially generated) events mapped to their exact locations. Events are distinguished by disease status and whether they are newly identified in a cluster exceeding the signaling threshold. Disease status may be replaced with any categorical variable included in the case input file.

A legend that distinguishes events as being *Inside Cluster, new entry*; *Inside Cluster, not new entry*; or *Outside Clusters* exceeding the RI signaling threshold can be displayed. Events are also distinguished as *Recent* versus *Prior* to a date specified on the *Recent Events* time slider in the HTML file, by default the start date of the most likely cluster (Figure 3; refer to the Visualizing Clusters and Cases on a Map: Supplement section in Multimedia Appendix 1).

### Drilldown Analysis

**Figure 4:**
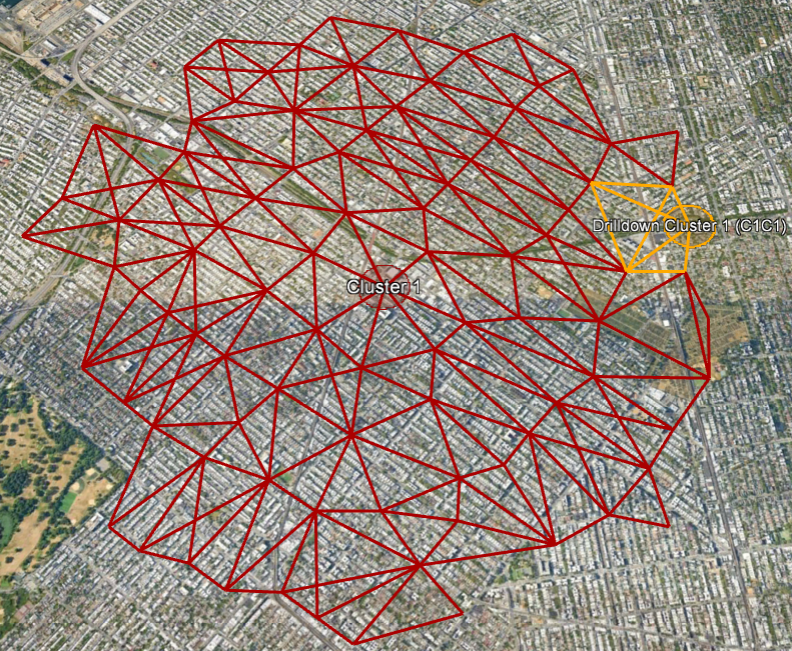
Google Earth visualization of drilldown cluster C1C1 (in orange) identified within cluster 1. The drilldown analysis is one tool for identifying smaller spatial clusters to help focus investigation efforts and resources.

Geographically large clusters can be further investigated using the SaTScan drilldown tool to determine whether events are randomly distributed or clustered within a cluster (Figure 4; refer to the Drilldown Analysis: Supplement section in Multimedia Appendix 1).

### Line List of Cluster Events

When a cluster is detected, SaTScan can produce a line list of all events in clusters exceeding the RI signaling threshold with information such as age, gender, and location of each event. This may help with determining whether an outbreak investigation is warranted, as well as with the investigation itself (refer to the Line List of Cluster Events: Supplement section in Multimedia Appendix 1).

Examining the line list is also an opportunity to check for data quality issues and other unintended drivers of signals, such as duplicate individuals, erroneous addresses, multiple events in the same household, events that do not ultimately meet the surveillance case definition, or events for which it can be determined that an available address is unrelated to an exposure site (eg, a person experiencing homelessness whose address is a hospital). Because the purpose of the system is to detect ongoing community clusters of disease, an analysis may be rerun excluding these events to determine whether the community cluster persists. At the BCD, we proactively check for and correct data quality issues (refer to the Requirements and System Setup section), but inspecting the line list can detect problems missed by other routine checks.

In addition, the line list may be useful for filtering detected clusters by the number of events with certain statuses, for example, requiring a minimum number of *confirmed* or *probable* cases to investigate. At the BCD, although we scan for clusters with as few as 2 events, we normally require ≥3 *confirmed*, *probable*, *suspected*, or *pending* events to warrant further investigation. Thus, producing a line list that includes a case status field can support cluster prioritization.

### Automated Email Alerts

The email alert feature can be used to automatically notify up to 2 groups of recipients of analysis results. One email can notify that the analysis has been completed, irrespective of the results, and another email can notify the same or a different group of recipients when a cluster exceeds the RI signaling threshold (refer to the Email Alerts section in Multimedia Appendix 1).

## Cluster Investigation and Response

A well-developed cluster detection system focuses staff attention on emerging outbreaks to catalyze field investigations [53]. At the BCD, disease experts interpret cluster output and determine whether to launch an investigation and response, which, depending on the disease, might involve prioritizing patient interviews, conducting environmental investigations (eg, inspecting cooling towers or food service establishments), and conducting community outreach and education to rapidly identify outbreak sources and interrupt ongoing transmission.

## Discussion

### Overview

This tutorial is intended to support public health officials in understanding the details of the successful communicable disease outbreak detection system in NYC [2,12-14] and how to adapt and fine-tune a similar system for their own jurisdictions. For early outbreak detection, spatiotemporal methods are preferable to purely temporal methods (eg, the refined historical limits method [42] and other time-series analyses) because, if data are analyzed at one resolution (eg, by county and week) while an outbreak is emerging at a different resolution (eg, a collection of a few census tracts over a few days), the outbreak may be difficult to detect. Unlike other software for spatiotemporal cluster detection (eg, the ArcGIS Emerging Hot Spot Analysis tool [54]), SaTScan analyzes data using scan statistics to search flexibly over time and space rather than within arbitrary administrative boundaries while accounting for multiple comparisons. SaTScan also avoids the modifiable areal unit problem [55] when using input data at the finest geographic resolution available, allowing for more precise identification of areas with elevated rates, and has options for spatial adjustments and nonparametric temporal adjustments to account for jurisdiction-wide trends. SaTScan version 10.1 includes multiple enhancements allowing for increased customizability, automation, and output visualization, which have not previously been described in real-world applications. Assuming input data are timely, complete, and accurate, the primary limitation of the system is diminished power to detect spatiotemporal outbreaks involving very few patients or involving patients located in a long and narrow area, such as along a river [56].

### Health Equity

Our cluster detection system supports the NYC Department of Health and Mental Hygiene’s mission to protect and promote the health of all New Yorkers. Many communicable diseases disproportionately affect residents of high-poverty areas [57]. The faster that public health officials can detect outbreaks anywhere, the sooner disease transmission can be interrupted to support health equity and harmful environmental exposures can be remediated. To minimize the number of patients excluded from spatiotemporal analyses, we maintain data quality by proactively correcting typographical errors in addresses failing to geocode and assigning persons who are unsheltered and without a geocodable address to a separate category. By selecting the space-time permutation probability model for our base analyses, we account for purely spatial patterns, such as areas with comparatively lower access to care and testing for reportable diseases. Nevertheless, if very few patients in an outbreak access care (eg, as a consequence of systemic racism), then outbreak detection may be delayed or missed entirely. Hence, better and wider access to health care will not only benefit those patients but also strengthen public health surveillance and response efforts that benefit all residents.

### Future Directions

Going forward, we advocate for more centralized analytic technical support for outbreak detection for state, tribal, local, and territorial public health departments in keeping with the Data Modernization Initiative [58] and other public health system investments. We hope to provide video tutorials to orient new users and foster a community of users to share knowledge and best practices. While we believe our recommendations should be generalizable to other jurisdictions in the United States and internationally, our practical experience is limited to monitoring certain reportable communicable diseases in one dense, urban area. To better understand generalizability, additional jurisdictions will similarly need to add their voices to this conversation and contribute their experiences using this system. We are particularly interested in the experiences of jurisdictions covering larger geographic and rural areas; experiences using the network locations file with user-specified, non-Euclidean distances; and the adaptation of this system for other data streams for infectious diseases, including syndromic surveillance, social media, wastewater, antimicrobial susceptibility testing, and veterinary data.

## Appendix

Multimedia Appendix 1 Further details about parameter settings and guidance for advanced options, output interpretation, and strategies for fine-tuning an analysis.

Multimedia Appendix 2 Video that demonstrates how to set up a cluster detection system and sample input and output files.

## Abbreviations

BCD: Bureau of Communicable Disease
NYC: New York City
RI: recurrence interval
