# Supplementary material for: Prospective Spatiotemporal Cluster Detection Using SaTScan: Tutorial for Designing and Fine-Tuning a System to Detect Reportable Communicable Disease Outbreaks
Source: JMIR Public Health Surveill. 2024 Jun 11;10:e50653. doi: 10.2196/50653 (PMC11200039; doi:10.2196/50653)
Supplement: Multimedia Appendix 1 [file publichealth_v10i1e50653_app1.pdf]

Multimedia Appendix 1 for *Prospective Spatiotemporal Cluster Detection Using SaTScan: Tutorial for Designing and Fine-Tuning a System to Detect Reportable Communicable Disease Outbreaks*

Here we provide further details about parameter settings introduced in the main text and guidance for advanced analysis options. We also demonstrate output interpretation using artificial data and introduce strategies for fine-tuning an analysis if it consistently produces results that are unsatisfactory and data quality issues have been ruled out as a cause.

Analysis Design: Supplement

In the Bureau of Communicable Disease (BCD) at the New York City (NYC) Department of Health and Mental Hygiene, we run the base analysis to detect unusual clusters of 34 reportable diseases. We use a 30-day maximum temporal window for amebiasis, babesiosis, campylobacteriosis, chikungunya virus disease, cholera, cryptosporidiosis, cyclosporiasis, dengue virus infection, encephalitis, invasive group A *Streptococcus* disease, invasive group B *Streptococcus* disease, giardiasis, invasive *Haemophilus influenzae* disease by biotype, human granulocytic anaplasmosis, human monocytic ehrlichiosis, hemolytic uremic syndrome, legionellosis, malaria, meningococcal disease, mpox, invasive *Streptococcus pneumoniae* disease, rickettsialpox, Rocky Mountain spotted fever, shigellosis, noncholera *Vibrio spp.* infection, vancomycin-intermediate *Staphylococcus aureus* infection, West Nile virus, and yersiniosis. We run additional analyses restricting to young children (aged <5-years) for amebiasis, cryptosporidiosis, giardiasis, and shigellosis and restricting to school-aged children (aged 5–18-years) for group A *Streptococcus* disease. We extend the maximum temporal

window to 60 days for hepatitis A and for salmonellosis (overall, and by serotype at a 14-day lag to allow for laboratory subtyping), Shiga toxin-producing *Escherichia coli*, typhoid fever, and paratyphoid fever and to 120 days for listeriosis for consistency with PulseNet USA cluster definitions. At the NYC Department of Health and Mental Hygiene, surveillance for other acute infectious diseases (eg, vaccine-preventable diseases and sexually transmitted infections) are conducted by other bureaus.

For some diseases, we further modified the base analysis (Table S1) [2]. For example, to account for possible non-residential exposure sites for legionellosis, we use a multiple address analysis to detect clusters using all geocoded location data available (eg, work addresses collected during patient interviews) [30]. For COVID-19, we scanned for clusters of elevated test positivity rather than case counts because case ascertainment during the public health emergency was highly affected by testing variability [14].

Table S1. Aims and selected characteristics of prospective SaTScan analyses to detect and monitor unusual clusters using reportable disease data, implemented by the Bureau of Communicable Disease, New York City Department of Health and Mental Hygiene.

		**Prospective space-time probability model**	**Input Files**	**Parameter settings**
**Base Analysis**[1]				
	Detect clusters of disease in space and time [2]	Space-time permutation	• Case file includes all reported events, whether subsequently confirmed or not<br>• Event location is home address<br>• Date of interest is best	• Day-of-week by space interaction adjustment to account for geographic variation in the daily pattern of health care-seeking behavior.<br>• 1-year study period ending day before analysis

[1] The base analysis describes most routine SaTScan analyses of reportable diseases implemented by the Bureau of Communicable Disease.

			approximation of disease onset<br>• Geographic aggregation is census tract<br>• Coordinate file includes 1 row per census tract with geographic coordinates of census tract centroids<br>• Network file connects neighboring census tracts unless separated by natural barriers	• Scan for clusters between 2 and 30 days long, with extensions for certain pathogens, and with a minimum of 2 events<br>• Signaling threshold of recurrence interval (RI) ≥100 days
**Modified Analyses**[2]				
	Detect clusters of legionellosis in space and time using all available addresses [30]	Space-time permutation	SaTScan v10.1.2 and prior:<br>• Case and coordinate files use Event ID instead of census tract as the location ID<br>• Coordinate file includes coordinates for each address per event (home, work, secondary residence, other)<br>As of SaTScan v10.2:<br>• Case file includes 1 row per event, with Event ID as <Identifier>[3]<br>• Coordinate file as in base analysis<br>• Multiple locations file consists of <Identifier> (Event ID) and <Location> (census tract) columns to link to the case and coordinate files, respectively, with 1 row per unique census tract for all available addresses per event (home, work, secondary residence, other)	• Include observation if at least 1 of its locations is in the window
	Detect clusters of legionellosis events specifically around a cooling tower and in time [13]	Space-time permutation	• Additional grid file with all cooling tower coordinates to define centroids of scanning circles	• Maximum spatial cluster size of 1 km

[2] Input files and parameter settings for the base analysis apply to all other analyses unless otherwise specified.
[3] Text in <> refers to fields as they are labeled in the SaTScan user interface.

	Detect clusters of listeriosis in space and time	Space-time permutation		• Scan for clusters between 7 and 365 days long because exposure to point sources can be intermittent and incubation periods can be up to 70 days [28]<br>• Extend baseline to 3 years because rule of thumb is for study period to be at least 3 times as long as the maximum temporal window<br>• Increase time aggregation to 7 days to reduce run time and because daily precision in cluster start date could be less important for prolonged clusters<br>• No adjustment for day-of-week by space interaction because data are aggregated by week
	Detect clusters of locally acquired Zika virus infection in space and time	Space-time permutation		• Maximum spatial cluster size of 1 km reflects typical range of an *Aedes* mosquito [27], allowing for some additional distance to include residences of people moving around and coming into contact with the same infected mosquito
	Detect clusters of young children diagnosed with norovirus in space and time	Space-time permutation	• Case file includes only the most recent event in a household within 7 days, consistent with duration of infectiousness and incubation period to avoid clusters that are driven by household transmission<br>• Restrict to events among children aged <6 years	• Signaling threshold increased to RI ≥365 days because for community-based norovirus outbreaks, only moderate or strong clusters affecting young children are considered actionable.
	Detect shigellosis clusters overall, by species, and within different age groups	Space-time permutation	• Main case file includes events for all species and ages<br>• Additional analysis restricts to people aged <5-years<br>• Additional analyses restrict to shigellosis species, i.e., *S. sonnei*, *S. flexneri*, *S. boydii*, and *S. dysenteriae*	

			• Additional analysis that excludes previous events in the same household within 35 days, consistent with duration of infectiousness and incubation period	
	Detect space-time clusters of positive tests for SARS-CoV-2 [14], adjusting for test type (molecular and antigen) using multiple datasets	Discrete Poisson model with nonparametric temporal trend adjustment	• 3 case files that include positive molecular tests only, positive antigen tests only, and both a positive molecular and antigen test<br>• 3 corresponding population files that include people tested (with a positive or negative test result) by 1 or both test types<br>• Input files exclude patients residing in congregate settings to distinguish community-acquired transmission<br>• Case files include only the most recent case in a household within 14 days to avoid clusters that are driven by household transmission. Population files retain either the most recent positive test per household within 14 days or if none, the most recent test in a household within 14 days.	• Lag study period end date by 3 days because data are very incomplete on more recent days<br>• Scan for clusters between 14 and 21 days long because only clusters persisting for at least 2 weeks were considered actionable<br>• Use 63-day study period because rule of thumb is for study period to be at least 3 times as long as the maximum temporal window, and volatility of testing practices made older data less comparable<br>• Increase minimum case requirement when the volume of cases was large<br>• Multiple datasets option with Purpose=Adjustment<br>• Apply purely spatial adjustment if interested in areas with unusual increases even if the absolute level is low<br>• Consider relative risk restrictions to detect only high rate, smaller clusters
	Detect space/time clusters of COVID-19 hospitalization and/or death	Space-time permutation	• Mutually exclusive datasets for hospitalization and death (if case is in both death and hospitalization, then remove from hospitalization)	• 2 analyses, lagging study period end date by 4 and 14 days because variability in timeliness of reporting by facility or regional health information organization could skew results<br>• Scan for clusters between 14 and 28 days long<br>• Multiple datasets option with Purpose=Adjustment

	Detect purely temporal clusters of serotypes adjusting for overall seasonal trends of *Salmonella* [25]	Space-time permutation	• Coordinate file replaces <Location> with serotype and assigns arbitrary x/y coordinates to each serotype 10 units apart<br>• Case file uses serotype as <Location>	• Maximum spatial cluster size is a circle with 1-unit cartesian unit radius<br>• No adjustment for day-of-week by space interaction
	Detect space-time clustering of emerging SARS-CoV-2 variants	Bernoulli with nonparametric temporal trend adjustment	• Case file includes cases of COVID-19 with sequencing results as emerging variant of interest<br>• Control file includes all other sequenced cases of COVID-19	• Scan for clusters between 14 and 28 days long because only clusters persisting for at least 2 weeks were considered actionable<br>• Study period end date is most recent sequencing result date, and start date is 84 days prior

Bernoulli and Poisson Probability Models

In most situations, we recommend using the space-time permutation model. Given that this model automatically adjusts for both purely spatial and purely temporal variation, there is no advantage to using the Poisson denominator-based probability model unless there are large shifts in available population denominator counts during the study period, which is unlikely over a one-year study period. Even if the population is not stable, for example with refugee resettlement, sufficiently timely and accurate denominator data are not practically available; even high-quality United States Census population denominators are lagged in public availability and underrepresent persons living in institutional settings or recently constructed housing, migrants, undocumented persons, and persons experiencing homelessness.

In the rare situation that case ascertainment over both time and space is highly affected by testing variability, a space-time permutation analysis may pick up clusters due to increased testing frequency, such as was true for COVID-19 due to localized and temporary testing outreach [14]. We opted for a denominator-based probability model to detect COVID-19 clusters, defining the denominator as the number of laboratory tests, while the case file was

restricted to positive tests. This was possible because temporary emergency regulations required laboratories to report negative test results [31]. By searching for areas with excess case counts while adjusting for the number of persons tested, areas where the proportion of positive laboratory tests increased faster than the citywide trend could be detected regardless of whether the local laboratory-based testing rate was low or high. A limitation of this approach was that as other respiratory pathogens (eg, influenza, respiratory syncytial virus) reemerged after low circulation during the early COVID-19 period, areas might have had low SARS-CoV-2 test positivity not because SARS-CoV-2 transmission was low, but because people seeking testing for COVID-19–like illness were infected with a different pathogen [32].

The Bernoulli probability model is preferred for Bernoulli-type data such as positive and negative SARS-CoV-2 test results, but the Bernoulli model has fewer SaTScan options for spatial and temporal adjustments than the Poisson model. Thus, to detect COVID-19 clusters, we used the Poisson model [14], which is a good approximation for Bernoulli-type data when there are few cases compared with controls (i.e., cases generally <10% of persons tested, which was typical for SARS-CoV-2 test positivity in NYC until the widespread adoption of home-based antigen testing) and can be used instead when these adjustments are needed (Figure S1). When the number of cases per people tested is not rare, the Poisson probability model is a poor approximation of the Bernoulli model, and clusters will be harder to detect.

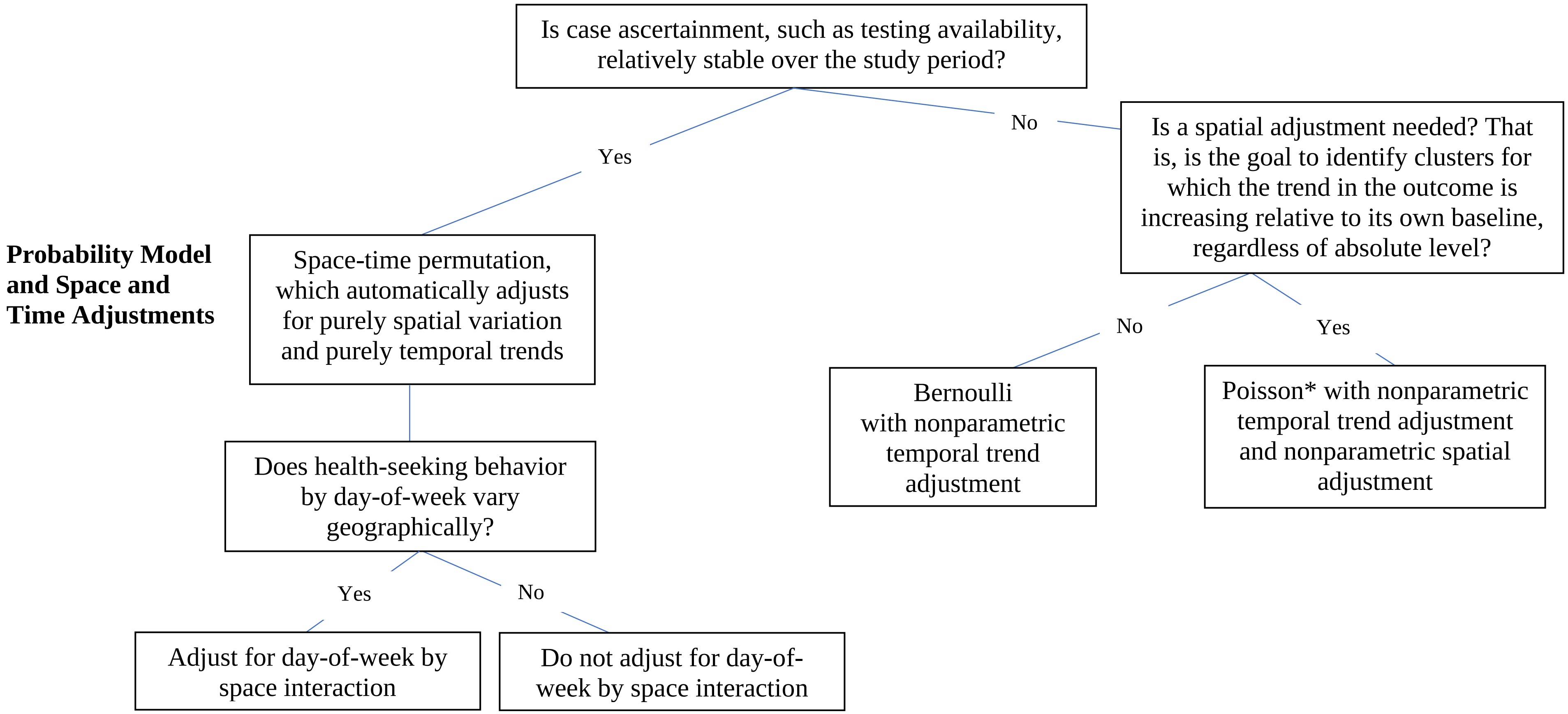

Figure S1. Decision tree encompassing the subset of prospective space-time probability model and spatial and temporal adjustment options that the Bureau of Communicable Disease at the New York City Department of Health and Mental Hygiene has found useful when designing analyses during 2014–2023 to detect emerging disease clusters using reportable disease data.

* If the number of events per population (or people tested) is not rare, then the Poisson probability model is a poor approximation of the Bernoulli model, and clusters will be harder to detect.

We used the Bernoulli probability model to detect spatial clustering of emerging SARS-CoV-2 variants. To adjust for the spatial distribution of patients whose isolates are sequenced, a particular variant defined the cases while all other variants were the controls. Because this analysis focused on newly emerging variants, such that the prevalence of the variant of interest was consistently low in all areas during the baseline period, this analysis did not require purely spatial adjustment.

Space and Time Adjustments: Supplement

We adjust for citywide purely temporal trends in all analyses. Adjusting for purely temporal trends is important when reporting volume is affected by external factors, such as seasonal variation or decreases in diagnoses for reportable communicable diseases during the emergency phase of the COVID-19 pandemic, which reflected both reduced exposure (less international travel, restaurant closures, social distancing, etc.) and reduced case ascertainment (less health care seeking, increased use of telehealth so fewer specimens were collected for laboratory-based testing, etc.) [33].

The Bernoulli and Poisson models do not automatically adjust for temporal trends. For the Poisson probability model, temporal trends can be adjusted for either nonparametrically or parametrically. With the latter, a log-linear adjustment can be user-specified, or a log-linear or log-quadratic adjustment can be automatically calculated using observed data. To decide which to use, we recommend plotting the overall data over time and using the corresponding automatically calculated parametric adjustment if the trend is roughly linear or roughly quadratic. More irregular temporal patterns are best modeled with the nonparametric adjustment, which is the only option available for the Bernoulli probability model. If the trend is actually log-

linear or log-quadratic but the nonparametric adjustment is applied, true clusters might be slightly harder to detect due to overadjustment. Short-term temporal trends in COVID-19 testing were often non-linear, so we opted for the flexibility and low maintenance of the nonparametric temporal trend adjustment for COVID-19 analyses.

For the Poisson model, it is also possible to adjust for purely spatial variation nonparametrically. Applying this adjustment will identify clusters where case counts or test positivity is increasing faster or decreasing slower than elsewhere and relative to the cluster area's baseline, even if the absolute level in the area is lower. For COVID-19, for example, adjusting for purely spatial clusters can provide awareness about localized, rapid increases in test positivity that could indicate areas with an emerging variant where specimens might be prioritized for whole-genome sequencing. When the aim instead was to allocate resources to areas with currently high test positivity, we did not apply a spatial adjustment. For this purpose, we inspected cluster temporal graphs and prioritized clusters with increasing trends during the cluster temporal window.

Control and Population Files

The Bernoulli and Poisson probability models require a control file and a population file, respectively. Each has the same format as the case file, with a location ID, a date, and the count of controls (eg, cases with sequencing results other than the SARS-CoV-2 variant of interest) or count of population at risk (eg, persons with SARS-CoV-2 testing). If for a particular location and date the population at-risk is zero (eg, a test positivity analysis with zero persons tested), it can be excluded from the Bernoulli model control file but must be included in the Poisson model population file specified as zero. If not, SaTScan will estimate the population for that location

and date through linear temporal interpolation, inflating the expected counts and potentially failing to detect a cluster around that location.

Multiple Data Streams

For some diseases, there may be 2 or more complementary data streams of different quality, severity, or type. In SaTScan, it is possible to specify multiple input files and search for clusters in all datasets simultaneously, applying an adjustment. We applied this adjustment when scanning for clusters of COVID-19 hospitalizations, deaths, or hospitalizations that resulted in death, which were different measures of COVID-19 severity.

To detect increasing COVID-19 transmission, we used laboratory-based results for both molecular and antigen tests, which had differential sensitivity/specificity and uptake across geography and time. Rather than treating the test types equally by summing them together, we generated 3 case files daily: (1) positive molecular tests only, (2) positive antigen tests only, and (3) both positive test types. Each file had 1 row per positive test and specimen collection date. If a person had >1 positive test per calendar week, we retained the first. The 3 corresponding population files were: (1) persons who received only molecular tests, (2) persons who received only antigen tests, and (3) persons who received both molecular and antigen tests. Each file had 1 row per census tract and date. For each week, if a person had a positive test, the first positive test was retained, and if not, the first negative test was retained. For days and census tracts where zero persons were tested, the population file specified the count as zero.

We ran a test-based analysis rather than a person-based analysis because when weekly occupational testing was mandated, many people received multiple laboratory-based tests within a short period of time. Keeping only the most recent negative test result per person during the

study period would have driven down recent test positivity compared with the baseline period, making it harder to detect an emerging cluster. As occupational testing mandates were removed, the test-based analysis became more similar to a person-based analysis.

While not yet used by the BCD, we plan to develop multivariate analyses with multiple data streams to detect clusters in 1 or more of them. For example, we plan to analyze syndromic data streams of emergency department visits for diarrhea together with reportable disease data streams for salmonellosis, campylobacteriosis, and other enteric pathogens [34]. A detected cluster could consist only of diarrhea, of diarrhea and salmonellosis, or of any other combination of data streams. Simultaneously analyzing non-specific but timely syndromic surveillance data together with less timely but specific, laboratory-based data could result in earlier outbreak detection.

Network File: Supplement

An enhancement in SaTScan v10.0 is the advanced option to scan locations along a network. One can use a network file to allow clusters to form around natural barriers such as bodies of water or mountain ranges or to form within communities that are geographically distant but linked by commuting patterns or a shared ethnicity or language. For example, portions of northeastern Queens and southwestern Brooklyn are geographically distant but connected through large populations of Chinese New Yorkers [35]. One could also conduct a health care facility-level analysis, connecting facilities with distances reflecting patient transfer volumes.

To use this feature, a network file must be created, either manually or using freely available R code [36], in which each row is a pair of locations that are directly connected. SaTScan can calculate the Euclidean distance between the pairs using the coordinates file, the

user can manually specify a distance between each pair (eg, travel time), or one can utilize a combination of the two (eg, geographically distant but linked communities could be connected with a user-specified distance shorter than the Euclidian distance while all other connections default to the Euclidean distance). A pair of locations without a direct link can only appear in the same cluster through intermediate locations along the network. In the BCD, we remove direct links across barriers like rivers in places without bridges, cemeteries, zoos, and airports using the MAF/TIGER Feature Class Codes specified in the United States Census Bureau area landmark file [36, 37] (Figure S2). If it is ambiguous whether 2 areas should be directly linked, we recommend erring on the side of more connectivity so as not to miss clusters. For quality control, we recommend visualizing the whole network to confirm that links are consistent with local knowledge about connectivity (Figure S2).

The network may include "islands" specified as a row with a location but no pair, which can represent true islands with limited connectivity or figurative islands of patients who would otherwise be excluded from analyses. We use this technique to detect emerging outbreaks affecting unsheltered persons experiencing homelessness by assigning them to a unique, artificial census tract, which we include as a row with no pair in the network file.

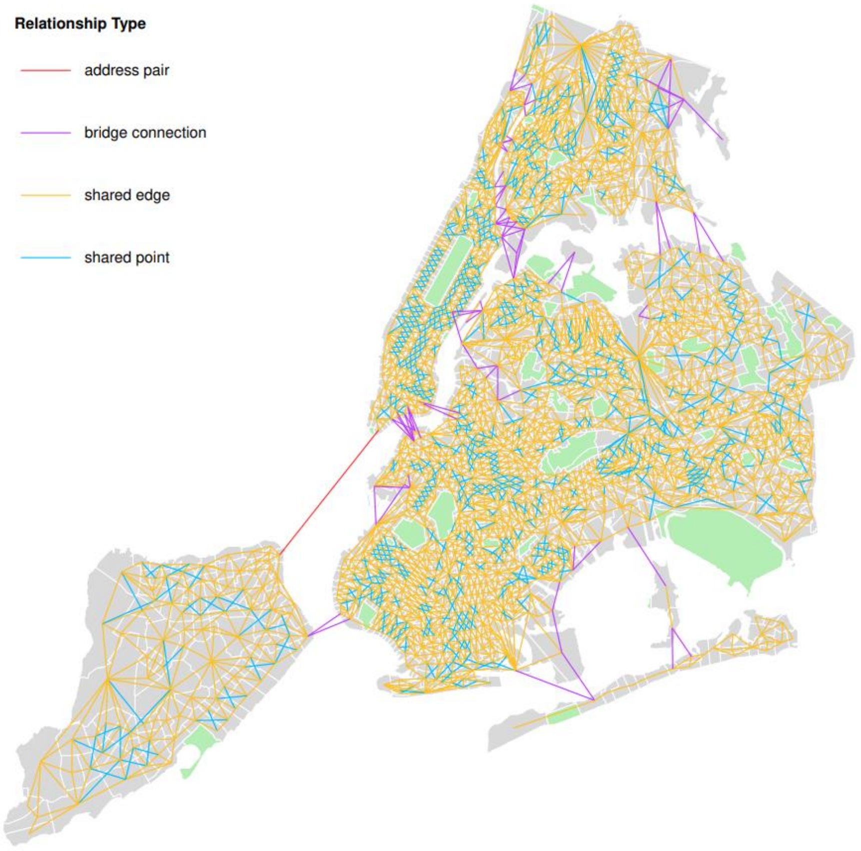

Figure S2. Visualization of connectivity across 2,125 census tracts in New York City, per Census 2010 boundaries. Each pair of census tracts connected by a shared edge, shared point, bridge, or other address pair (eg, census tracts connected by the Staten Island Ferry) is specified in the network input file. Euclidean distances between census tract pairs are calculated by SaTScan using the coordinates file. Uninhabited areas (eg, airports) are displayed in green.

Video Demonstration

In an accompanying video in Multimedia Appendix 2, we demonstrate how to set up a cluster detection system. We show how to read in .csv or .txt input files using the File Import Wizard and select the “Save these settings and read directly from file source when running analysis” option. We also provide overviews on setting up a network file, automating multiple

analyses and email alerts, and using built-in output features including line lists, maps, and temporal graphs. Additional video tutorials to orient new users will be provided at satscan.org.

Cluster Output Interpretation

We now present and interpret sample output, including HTML and KML maps as well as temporal graphs and a line list of cluster events for each cluster exceeding the signaling threshold of RI $\geq$100 days. We investigate a large cluster using the Drilldown feature and set up automated email alerts.

Provided in Multimedia Appendix 2 are sample input files, using actual NYC coordinates but artificially created case data. Running this sample space-time permutation analysis in SaTScan, which uses our base analysis parameter settings, will produce the output files provided and described below, allowing the reader to follow along and gain hands-on experience interacting with output files to interpret clusters.

Main Text-Based Results File

An abbreviated standard text-based results file is shown in Figure S3 (refer also to the FakeOutput.txt output file). Analysis summary information is followed by detected clusters listed in order of decreasing test statistic.

```
Prospective Space-Time analysis
scanning for clusters with high rates
using the Space-Time Permutation model.
Adjust for weekly trends nonparametrically.
______________________________________________________________________________

SUMMARY OF DATA

Study period.......................: 2021/01/02 to 2022/01/01
Number of locations................: 2125
Total number of cases..............: 2530
______________________________________________________________________________

CLUSTERS DETECTED

1.Location IDs included.: 36047025000, 36047024200, ... [90 more]
  Coordinates...........: (40.623626 N, 73.995838 W)
  Span..................: 4.50 km
  Time frame............: 2021/12/12 to 2022/1/1
  Number of cases.......: 30
  Expected cases........: 6.81
  Observed / expected...: 4.40
  Test statistic........: 21.391220
  P-value...............: 0.00000016
  Recurrence interval...: 17007 years

2.Location IDs included.: 36047049600, 36047049800, ... [74 more]
  Coordinates...........: (40.643687 N, 73.980438 W)
  Span..................: 4.70 km
  Time frame............: 2021/12/12 to 2022/1/1
  Number of cases.......: 21
  Expected cases........: 5.34
  Observed / expected...: 3.93
  Test statistic........: 13.134523
  P-value...............: 0.0011
  Recurrence interval...: 2.5 years

3.Location IDs included.: 36047093400, 36047093600, ... [29 more]
  Coordinates...........: (40.648924 N, 73.919015 W)
  Span..................: 3.07 km
  Time frame............: 2021/12/24 to 2022/1/1
  Number of cases.......: 9
  Expected cases........: 0.89
  Observed / expected...: 10.16
  Test statistic........: 12.767783
  P-value...............: 0.0016
  Recurrence interval...: 1.7 years

4.Location IDs included.: 36081094202, 36081094203, 36081095400, 36081094201, 36081096400
  Coordinates...........: (40.590185 N, 73.809188 W)
  Span..................: 2.54 km
  Time frame............: 2021/12/23 to 2022/1/1
  Number of cases.......: 8
  Expected cases........: 0.68
  Observed / expected...: 11.84
  Test statistic........: 12.458558
  P-value...............: 0.0023
  Recurrence interval...: 1.2 years
```

Figure S3. Results file excerpt showing the 4 most likely clusters detected using artificial data.

Data do not represent real disease events.

The most likely cluster consisted of 92 census tracts, with 30 events occurring within a 21-day period, when 6.81 were expected by chance, for an observed / expected ratio of 4.40. The "Location IDs included" are listed in order of centrality, such that the first listed census tract is at the center of the cluster. The geographical span is 4.50 km, which is the distance between the 2 furthest locations in the cluster. This cluster is extremely unlikely to have occurred by chance (RI=17,007 years). If we continued daily analysis for 17,007 years, then under the null hypothesis of there never being an outbreak for this disease, the expected number of false clusters seen at this magnitude or higher is one.

This RI threshold acts less as a binary measure of whether a cluster is concerning and more as a trigger for human review, considering not only how statistically unusual the cluster is, but also the observed event count, observed/expected ratio, epidemic curve trajectory, geographic extent, demographic composition, and persistence. Had this been a real analysis, BCD staff would examine the other output produced by SaTScan for this cluster and initiate an epidemiologic investigation.

Temporal Graphs: Supplement

Temporal graphs are useful for visualizing epidemic curves (refer to Figure 2 in main text and FakeOutput.temporal.html output file). Each time series can be toggled on and off, and the graph can be zoomed in on the x- or y-axis. During the cluster period, the observed events are consistently higher than the number of expected events, shown in dark green. Rolling over each date displays the exact number of events observed and expected.

The trend during the cluster period is increasing, which suggests that this cluster is ongoing, and investigators may choose to prioritize this over a cluster with a decreasing trend.

Visualizing Clusters and Cases on a Map: Supplement

We selected the *HTML file for Google Maps* or *KML file for Google Earth* output options, ensuring the coordinates file includes latitude/longitude rather than x/y coordinates, to produce maps for visualizing the spatial extent of clusters. This analysis found 4 clusters citywide, including 2 overlapping clusters in southwest Brooklyn (refer to Figure 3 in the main text and FakeOutput.clustermap.html output file).

Event locations can be overlaid and grouped by categorical variables included in the case input file, such as disease status (Figure 3), by formatting the case file so there is 1 row per event with its <Descriptive Latitude> and <Descriptive Longitude> coordinates indicated in the Import File Wizard (accessed by clicking on the […] button next to the Case File on the Input tab). These coordinates are not used in the analysis and are for visualization purposes only.

A legend distinguishes which events are new and inside clusters. If no cache file is specified, all cluster events will be labeled as "Inside Cluster, new entry." The HTML output has more flexibility than the KML output to select or adjust the categories displayed. For example, events can be restricted to a subset of disease statuses and a subset of age groups by specifying these choices in the "Display Events By:" and "Exclude Events:" dropdown menus.

When the time slider depicting events in the period in the HTML file or at the top of the Google Earth map is played, events appear on the map in the order they occurred and can be selected to display summary information. In the HTML output, the user can exclude events before a specific date by moving the left-hand side of the slider.

Google Earth output is better suited for visualizing overlapping primary and secondary clusters or overlaying output files for comparison, which is useful when using the Drilldown

tool. To differentiate between overlapping clusters, select a contrasting color and different line thickness or opacity for each overlapping cluster [right click “Cluster X Edges” > Properties > Style,Color tab in the Places Pane]. In addition to referring to the main text-based results file for the geographical span of a cluster in kilometers, its size can also be approximated using the ruler in the Google Earth toolbar and selecting 2 points on opposite ends of the cluster to measure the distance between them.

Sometimes a cluster exceeding the RI signaling threshold will expand geographically over time. As the cluster expands, it may not only incorporate new, recent events but also older events that are within the expanded geographical area of the expanded cluster. Those older events are generally less worthwhile to include in a cluster investigation.

Drilldown Analysis: Supplement

Because Cluster 1 was geographically large, we ran a drilldown analysis to determine whether its events were evenly distributed or clustered within the cluster. For space-time analyses, the drilldown tool restricts the input data to the geographic extent of the cluster. Within this smaller extent, one can either apply the same design as the main analysis or a purely spatial analysis using the Bernoulli model, where “cases” are cases during the cluster period and “controls” are cases before the cluster period. We recommend drilling down both ways and inspecting clusters identified using either method.

One or more drilldown levels within clusters may be identified; the first level focuses on areas within the original cluster (eg, drilldown Cluster 1 within original Cluster 1 [C1C1]), the second level further focuses within those clusters (eg, drilldown Cluster 1 within drilldown Cluster 2 within original Cluster 3 [C3C2C1]), and so on. The drilldown analysis identified a

cluster exceeding the RI signaling threshold (C1C1) within Cluster 1 (refer to Figure 4 in main text). Events within C1C1 may be prioritized for investigation over other events in the larger cluster.

An analysis may also be repeated on an ad-hoc basis by applying a maximum reported spatial cluster size, which limits output to report only those clusters that are smaller than the specified maximum *reported* size while the inference is still based on the maximum cluster size *evaluated*. Technically, this is done by limiting the cluster size more for the real dataset while keeping the original maximum size when doing the calculations for the random simulated datasets. We typically use this approach in addition to a Drilldown analysis as both may detect clusters within larger clusters. If an analysis consistently detects clusters that are too large, refer to the Clusters Are Consistently Large and Uninteresting section below, which details strategies for fine-tuning input data and parameter settings.

Line List of Cluster Events: Supplement

If the case file is formatted so there is 1 row per event and the user specifies in the Import File Wizard that it contains line list information, SaTScan will produce a line list in csv format containing the specified characteristics of events in clusters exceeding the RI signaling threshold (Figure S4 and FakeOutput.linelist.csv output file). These characteristics, which can include, for example, a unique identifier, demographic characteristics, and street address (rather than aggregating to the area's centroid), are not used in cluster analysis.

Line lists can distinguish whether events are newly added to a cluster. If an <Individual> variable (eg, event ID) is specified in the line list tab of the Import File Wizard, a cache file will be created the first time a recurring analysis is run. Pointing to this file in subsequent runs will

prompt SaTScan to compare events in current clusters with those in clusters identified previously (refer to event_cache.txt input file). If an event ID does not appear in the cache file, it will be labeled as new in the line list and as a *new entry* on the map.

Events are labeled as primary or secondary, where secondary events also appear in a stronger overlapping cluster. Filtering the line list to hierarchy = *Primary* will result in a list of unique events.

The line list may be used in combination with other output to determine whether further investigation is warranted. For example, the results file and map output (Figure S3) show that Cluster 3 is geographically focused with a span of 3.07 km, and the line list reveals that all events are newly added and among children (Figure S4). In contrast, Cluster 4 is an ongoing cluster (eg, ≥1 event appeared in previously identified clusters) with no new events. The RI for Cluster 4 decreased compared with the previous day, and there are no common demographic characteristics. Thus, investigation of events comprising Cluster 3 may be prioritized over Cluster 4.

Cluster	Hierarchy	New Event	EventID	event_date	EventLongitude	EventLatitude	age	disease_code	disease_status_final	gender
3	Primary	New	92	12/26/2021	40.65459326	-73.90178348	15	XXX	PROBABLE	MALE
3	Primary	New	301	12/31/2021	40.6542727	-73.93223049	13	XXX	PROBABLE	MALE
3	Primary	New	572	12/25/2021	40.65494973	-73.92032745	15	XXX	CONFIRMED	MALE
3	Primary	New	812	12/24/2021	40.6602761	-73.91648005	13	XXX	CONFIRMED	MALE
3	Primary	New	1365	12/27/2021	40.63995929	-73.93026398	12	XXX	CONFIRMED	FEMALE
3	Primary	New	2530	1/1/2022	40.66191449	-73.92493123	14	XXX	PROBABLE	MALE
3	Primary	New	2531	1/1/2022	40.66107386	-73.92337963	17	XXX	PENDING	FEMALE
3	Primary	New	2536	1/1/2022	40.64455893	-73.93570566	17	XXX	PENDING	FEMALE
3	Primary	New	2537	1/1/2022	40.66162087	-73.92390253	12	XXX	PENDING	FEMALE
4	Primary		2521	12/31/2021	40.59780253	-73.80001219	78	XXX	PROBABLE	MALE
4	Primary		2522	12/28/2021	40.58592727	-73.82138333	59	XXX	CONFIRMED	MALE
4	Primary		2523	12/28/2021	40.59836294	-73.79423959	74	XXX	CONFIRMED	MALE
4	Primary		2524	12/23/2021	40.5872941	-73.80526836	9	XXX	CONFIRMED	MALE
4	Primary		2525	12/27/2021	40.59220138	-73.7983795	41	XXX	CONFIRMED	MALE
4	Primary		2526	12/30/2021	40.58865607	-73.79141191	11	XXX	CONFIRMED	FEMALE
4	Primary		2527	12/31/2021	40.5870776	-73.80831095	8	XXX	CONFIRMED	FEMALE
4	Primary		2528	12/27/2021	40.59498999	-73.80213736	38	XXX	CONFIRMED	MALE

Figure S4. Sample line list for 2 clusters. Cluster 3 is composed of all new events with a narrow age range, while Cluster 4 does not include any new events and patients have no apparent demographic similarities.

Email Alerts

SaTScan can send automated email alerts with a summary of the analysis results (Figure S5) to 2 groups of recipients through mail server settings defined in Preferences and Settings. We chose to attach the text-based results file to the email, and we used tags (eg, <date>, <signal-text>, <output-directory>) to include the date of the analysis in the subject line and a list of identified clusters and a link to the location of the result file in the body of the email.

If a cache file is used, email alerts can distinguish new versus ongoing clusters. If composed entirely of events that are not in the cache file, a cluster will be labeled new in the body of the email. Otherwise, it will be labeled as ongoing.

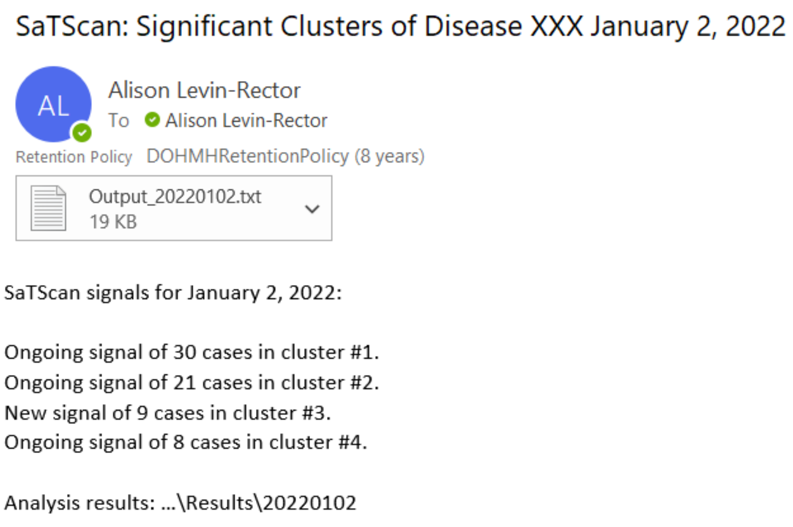

Figure S5. Example email alert notifying recipients of clusters exceeding the signaling threshold.

System Fine-Tuning

If an analysis consistently produces results that are unsatisfactory and data quality issues have been ruled out as a cause, then the input files or parameter settings may need fine-tuning. What follows are issues we have encountered while developing and monitoring BCD analyses, along with tools and techniques to address them.

Missed or Delayed Outbreak Detection

The system is not designed to detect outbreaks that do not have a strong geographical component, such as those caused by a widely disseminated contaminated commercial product, or by an exposure at a point source location, such as a concert or a tourist site. However, if known acute, localized clusters are not signaling, consider fine-tuning the system set-up.

In addition to automating and maintaining electronic disease reporting and geocoding processes to ensure uninterrupted, timely, and complete data feeds, parameter settings or input data may need adjustment to successfully and quickly identify disease outbreaks.

Difficulty detecting small outbreaks or outbreaks at the boundaries of geographic units

The less data are spatially aggregated, the more precisely and quickly areas with elevated rates can be identified. If data are aggregated into larger geographic units, such as ZIP codes or counties rather than census tracts, clusters may include large expanses where there are few cases, and outbreaks at the boundaries of geographic units may be missed.

Difficulty detecting outbreaks affecting a particular age group

If clusters affecting specific age groups are of particular concern, add age-restricted subgroup analyses. For example, for amebiasis, cryptosporidiosis, giardiasis, and shigellosis, we analyze children aged <5-years separately to detect outbreaks that might affect patients attending child care programs.

Difficulty detecting outbreaks where people are exposed far from their home

Outbreaks can affect persons working or spending time in a specific area, even if they do not live there. Typically, the patient's home address is the most accessible address for analysis, but SaTScan can incorporate multiple locations for the same person, such as both a home and a work address. That person will then be part of a cluster if either the home or work address is within the cluster.

For legionellosis, we also run a sensitivity analysis with multiple addresses per person, including work address, secondary residence, and any other address available, including addresses of local hotels where patients residing in other jurisdictions stayed, if notified by the Centers for Disease Control and Prevention [30]. We enable the advanced input feature to *include observation if at least one of its locations is in the window*. Functionality to search for clusters using multiple addresses per person along a network locations file is available as of SaTScan v10.2.

Difficulty detecting outbreaks because of missing data

Outbreaks may be missed when laboratories or health care providers fail to report events to the health department. To preempt this, we use SaTScan to monitor for laboratory drop-offs in

reporting, overall and by disease [26], and coordinate with the laboratories to resolve reporting issues. Additional monitoring should be conducted to ensure data completeness. If large sites fail to report, consider restricting the analysis to sites with consistent reporting throughout the study period.

To avoid missing outbreaks affecting patients who are experiencing homelessness and have a missing, inaccurate, or undefined spatial location, assign them to a unique, artificial census tract (refer to the Network file: Supplement section), so they can be included in analyses.

Difficulty detecting new outbreaks in areas with prior outbreaks

Historic outbreaks can interfere with the detection of subsequent outbreaks. For example, in a space-time permutation analysis, events in an historic outbreak will increase the expected event count for that area, making it harder to detect a new outbreak in the same area. Major historical outbreaks should be removed from the input files, in one of several ways.

For outbreaks caused by an exposure in a specific building or institution, such as an outbreak among residents of a nursing home, the outbreak-associated events can be excluded from the input file for subsequent analyses so as not to elevate the baseline. When major, community-acquired outbreaks occur, outbreak-associated events might be indistinguishable from background events that would have occurred even in the absence of the outbreak. Removing all these events from the baseline could be just as problematic as leaving them all in, by making the expected event count going forward in that area too low, which could cause false signals. For a space-time permutation analysis, the solution is to remove all events from the time period during which the historical cluster occurred, whether or not they were within the geographical boundary of the cluster. For a space-time Bernoulli analysis, the solution is to

remove not only all the events in the cluster but all the controls as well. For a space-time Poisson analysis, the solution is to remove all the events in the historical cluster and use the adjustment file to adjust for known relative risks, setting the risk to zero for the days and locations for which all the events were removed.

Difficulty detecting outbreaks affecting people who reside in areas not connected in the network file

If a pair of census tracts connected by disease transmission is omitted from the network file, SaTScan may miss clusters that span them. If it is ambiguous whether any 2 areas are directly linked, such as those separated by a small park, we recommend erring on the side of more connectivity so as not to miss clusters.

Difficulty detecting weak clusters that are potentially actionable outbreaks

Choosing the RI signaling threshold balances early detection with the positive predictive value of identifying a cluster of public health interest. Decreasing the RI threshold prioritizes early detection and will result in clusters that are more likely to have occurred by chance, while increasing it will lead to fewer false signals at the expense of timely cluster detection. In the base analysis, we chose an RI threshold of 100 days, but for norovirus among children aged <6 years, we chose a threshold of 365 days because weak clusters were not considered to be actionable. For COVID-19, we did not impose a cutoff and instead prioritized early detection, reviewing all clusters detected regardless of RI. If using an RI threshold for signaling ≥1 year and detecting weaker but potentially actionable clusters is a priority, consider decreasing the RI threshold.

Clusters Are Consistently Large and Uninteresting

If detected clusters regularly cover a geographical area that is too large to be useful, instead of repeating each analysis using the drilldown feature or limiting the maximum reported spatial cluster size on an ad-hoc basis, we recommend adjusting the inputs and parameter settings by for example setting a minimum relative risk or reducing the maximum spatial cluster size (refer to the Maximum Spatial Cluster Size section).

A user may be tempted to break up the jurisdiction into smaller areas and run independent spatiotemporal analyses for each area, but doing so would miss clusters spanning borders between areas. We recommend that large jurisdictions (eg, states, large counties) run one analysis and aggregate data to the smallest geographic unit available. Small jurisdictions (eg, small counties or cities) are best incorporated as part of statewide analyses because (1) if the cluster affects most of the area, it will not be detected when adjusting for purely temporal trends, (2) a small area is likely to be only part of an outbreak, making it more difficult to detect, (3) adjustment for multiple testing would be less satisfactory, generating more false alarms, and (4) it is logistically more efficient to run one geographically large analysis rather than dozens of smaller analyses.

Clusters span hard-to-cross boundaries

If large clusters span areas such as lakes, rivers, or mountain ranges, then the network file should be used to scan along a network. If a network file is being used, then local knowledge should be considered to determine whether links should be removed to reduce connectivity between populated areas separated by hard-to-cross boundaries.

Clusters with a relative risk near 1 are of limited public health interest

When the sample size is large, SaTScan has adequate power to detect clusters with a small effect size, eg, a relative risk close to 1. While exceeding the RI signaling threshold, such clusters may not be of public health importance. It is possible to restrict the analysis to detect clusters with a minimum relative risk, such as requiring clusters to have a relative risk ≥1.5. For COVID-19, we adjusted the minimum relative risk threshold depending on the surveillance needs (i.e., whether there was more operational interest in directing resources toward larger geographic areas with lower excess risk or toward smaller, more focused clusters with highly elevated risk).

Too Many Signals

SaTScan uses the maximum likelihood function to rank clusters in terms of their likelihood to have occurred by chance or not, but that is a purely statistical consideration. Some clusters may be dismissed as uninteresting based on additional criteria, such as epidemic curve trajectory, geographic extent, demographic composition, and persistence. If their volume is high, consider fine-tuning the system.

Clusters are driven by duplicated events or incorrect patient addresses

Proactively checking for and correcting data errors, such as duplicated events or incorrect patient addresses, can reduce false signals. Maintain quality assurance to ensure that multiple reports of the same event are correctly merged and to correct when laboratories mis-report patients' residential addresses.

Clusters are driven by the changing nature of input data

If data in the temporal window and the baseline period are not comparable over both space and time, clusters may be erroneously detected. If incomparability is the result of a change in surveillance, for example adoption of new testing practices, the study period should be shortened to exclude the time when the change occurred. We temporarily shortened the study period for nine reportable diseases when laboratories serving different parts of NYC adopted a culture-independent diagnostic test at different times, leading to increased case ascertainment [29]. If adoption of new testing practices occurs uniformly across a jurisdiction, it will be accounted for by adjusting for purely temporal trends (refer to the Space and Time Adjustments section).

Clusters are driven by reports that do not represent true illnesses

In the base analysis, we include events that are still pending investigation. This prioritizes timeliness but can result in signals that are driven by events that do not ultimately meet the surveillance case definition, and it may be necessary to use stricter inclusion criteria for analysis. For example, for their respective analyses, we exclude legionellosis events with only a positive antibody test and Shiga toxin-producing *Escherichia coli* events with only a positive culture-independent diagnostic test because they are likely to be reclassified as *not a case* upon investigation and were driving false signals. We also exclude events with only negative laboratory results from analyses.

If very few events of a disease are ultimately confirmed, we recommend excluding events pending case status classification and imposing a lag to allow for case investigations. For Rocky Mountain spotted fever, for example, because most pending events are ruled out as not cases, we

apply a 28-day lag and restrict to confirmed, probable, and suspected cases; similarly for Zika virus disease, we apply a 14-day lag and restrict to confirmed and probable cases.

Clusters are driven by within-household transmission

Over-signaling with community-acquired outbreaks can occur if a large proportion of person-to-person transmission occurs within households or buildings. For norovirus, shigellosis, and COVID-19, where household transmission is common, we restrict the case input file to include at most 1 event per household. We define a household as people living in the same building who share either a surname or an apartment number. This imperfect definition might fail to capture within-household transmission when apartment number is missing or misclassify as within-household transmission patients residing in the same building who share a surname but do not cohabitate. Nevertheless, this restriction improves the probability that detected clusters will not be driven by within-household transmission. We apply this restriction only if the onset dates of ≥2 events are within a predefined window based on the duration of infectiousness and incubation period that make secondary transmission likely for that disease. For norovirus, we use a 7-day window, for COVID-19, we use a 14-day window, and for shigellosis, we use a 35-day window. The BCD retains the most recent event rather than the first event in a household to focus attention on ongoing outbreaks.

Clusters represent weak outbreaks that are not actionable given available resources

If the RI signaling threshold is <1, increasing it can reduce the number of signals for weak, unactionable clusters.
