# Supplementary material for: Prospective Spatiotemporal Cluster Detection Using SaTScan: Tutorial for Designing and Fine-Tuning a System to Detect Reportable Communicable Disease Outbreaks
Source: JMIR Public Health Surveill. 2024 Jun 11;10:e50653. doi: 10.2196/50653 (PMC11200039; doi:10.2196/50653)
Supplement: Multimedia Appendix 2 [file publichealth_v10i1e50653_app2.zip › Multimedia Appendix 2/SpaceTimePermutation sample analysis/output files/FakeOutput.clustermap.html]

 


Cluster Map


Generated with SaTScan v10.2 Beta 5 Build 1

Display Clusters:

Cluster 1Cluster 2Cluster 3Cluster 4
Cluster 5Cluster 6Cluster 7

Toggle display of clusters.

High and low clusters
High only
Low only

Toggle display of clusters for scan rate.

Secondary Clusters:

Hierarchical
Gini

Display options for secondary clusters.

Show clusters using:

Circles/Edges
Locations

Display options for clusters.

Show all location points

Toggle display of location points.

Show all network edges

Toggle display of all network edges.

Display Individuals By:

CONFIRMED (1470)PENDING (558)PROBABLE (502)

Displays markers for selected groups.

Separate Icons:

Maximum of 8 can be distinguished by icon.

Exclude Individuals:

CONFIRMED (1470)PENDING (558)PROBABLE (502)

Filter to exclude markers of displayed individuals.

Individuals In Study Period

11/23/2021 to 3/2/2022

Run
Pause
Reset

Run Delay: 50 millisecondsRecent Individuals

Recent as of 11/23/2021

Display Individuals Legend

Individuals Icon Size

🠘 smallerlarger 🠚

Display Data:

Clusters

Cluster Locations

Total Locations

0 Total Individuals

Print

Print

### Legend
