# Supplementary material for: Prospective Spatiotemporal Cluster Detection Using SaTScan: Tutorial for Designing and Fine-Tuning a System to Detect Reportable Communicable Disease Outbreaks
Source: JMIR Public Health Surveill. 2024 Jun 11;10:e50653. doi: 10.2196/50653 (PMC11200039; doi:10.2196/50653)
Supplement: Multimedia Appendix 2 [file publichealth_v10i1e50653_app2.zip › Multimedia Appendix 2/SpaceTimePermutation sample analysis/output files/FakeOutput.temporal.html]

 


Cluster Temporal Graph


Search

Choose which graphs to display:

Cluster #1
Cluster #2
Cluster #3
Cluster #4

Apply
Cancel

0 % Complete

Show Chart Options

#### Chart Options

Title

Title can be changed by editing this text.

Observed Chart Type

Histogram

Line

Switch the series type between line and histogram.

Cluster Band

Show Cluster Band

Band stretching across the plot area marking cluster interval.

To zoom a portion of the chart, select and drag mouse within the chart. Hold down shift key to pan zoomed chart.

Close Chart Options

Show Chart Options

#### Chart Options

Title

Title can be changed by editing this text.

Observed Chart Type

Histogram

Line

Switch the series type between line and histogram.

Cluster Band

Show Cluster Band

Band stretching across the plot area marking cluster interval.

To zoom a portion of the chart, select and drag mouse within the chart. Hold down shift key to pan zoomed chart.

Close Chart Options

Show Chart Options

#### Chart Options

Title

Title can be changed by editing this text.

Observed Chart Type

Histogram

Line

Switch the series type between line and histogram.

Cluster Band

Show Cluster Band

Band stretching across the plot area marking cluster interval.

To zoom a portion of the chart, select and drag mouse within the chart. Hold down shift key to pan zoomed chart.

Close Chart Options

Show Chart Options

#### Chart Options

Title

Title can be changed by editing this text.

Observed Chart Type

Histogram

Line

Switch the series type between line and histogram.

Cluster Band

Show Cluster Band

Band stretching across the plot area marking cluster interval.

To zoom a portion of the chart, select and drag mouse within the chart. Hold down shift key to pan zoomed chart.

Close Chart Options

Generated with SaTScan v10.2 Beta 5 Build 1
